# Resting State Healthy EEG: The First Wave of the Cuban Normative Database

**DOI:** 10.3389/fnins.2020.555119

**Published:** 2020-12-01

**Authors:** Jorge Bosch-Bayard, Lidice Galan, Eduardo Aubert Vazquez, Trinidad Virues Alba, Pedro A. Valdes-Sosa

**Affiliations:** ^1^The Clinical Hospital of Chengdu Brain Sciences, University of Electronic Sciences and Technology of China, Chengdu, China; ^2^McGill Centre for Integrative Neurosciences MCIN, Ludmer Centre for Mental Health, Montreal Neurological Institute, McGill University, Montreal, QC, Canada; ^3^Cuban Neuroscience Center, La Habana, Cuba

**Keywords:** EEG normative databases, EEG quantitative analysis, EEG maturation, EEG cross-spectra, EEG resting state

## Introduction

Data sharing is one of the principal premises of open science. It is understood as one of the main sources for research reproducibility, data reuse and data integration. It is a good way of having well-documented datasets that guarantee data provenance which allow to reproduce results with different samples recorded under different conditions. There are currently two directions from where shared data come: one is from the big, international, multidisciplinary multimillionaire projects which produce and share enormous sets of data. The other one is the thousands of small datasets produced by a big number of small research groups all around the world, who produce very rich and diverse types of data. In the data sharing and open science argot, these datasets are called the long-tail data (Ferguson et al., 2014). In contraposition, the huge datasets created for those big international projects are called big data. Long-tail data convert numerous small datasets provided by different sources into big data to provide a better knowledge and understanding of a specific aspect of the brain functioning.

The present contribution falls into the category of the long-tail data. We share a dataset of resting state EEG collected at the Cuban Neurosciences Center, representative sample of normal subjects of the Cuban population. This sample was recorded between 1988 and 1990 in the Havana province. This province, the capital of the country, is quite representative of the whole Cuban population. The subjects were selected by a quasi-random procedure, which guaranteed an adequate age and sex representation along the whole spanned age range, which goes from 5 to 80 years old. The sample comprised 211 normal subjects (105 males, 106 females), who were selected under very strict clinical criteria, which rejected about 65% percent of the recruited sample. A very exhaustive clinical history was applied to the subjects, which accounted for a big number of possible medical conditions of family history of risk factors.

EEG recordings of resting state condition with Eyes Closed and Eyes Opened were carried out to all subjects. Expert electroencephalographers clinically evaluated the EEG recordings and rejected EEG with abnormalities under very strict criteria. However, subjects with EEG deviations like the absence of the Alpha peak, which is considered normal if not accompanied by other factors, were kept in the sample. Therefore, this a sample which is representative of the normal population, and not a sample of the “supernormal” population.

The original EEG recording contained 19 electrodes of the standard 10–20 positioning system, recorded at a sampling period of 10 msec. Here we list the used channels montage, in the same order as they appear stored in the files: Fp1, Fp2, F3, F4, C3, C4, P3, P4, O1, O2, F7, F8, T3, T4, T5, T6, Fz, Cz, Pz.

Twenty-four epochs of visually stationary, and artifact free EEG signals comprising 256 time points each, were carefully selected by visual inspection of the clinical electroencephalographers. However, due to storage limitations, the EEG time signals were not saved. The signals were converted to the frequency domain by means of the Fast Fourier Transform (FFT) and the EEG cross-spectral matrices for each subject were saved. For stationary data it is well-known that the cross spectral matrices retain all the statistical properties of the original signals.

Since the signals were assumed to be stationary, and the cross-spectral matrices were obtained by averaging 24 epochs of artifact free EEG activity, it is guaranteed that the FFT complex coefficients have an approximate complex Gaussian distribution at each frequency, and also they are independent between frequencies (Brillinger, 1974, 2001). Under these conditions, the Gaussian distribution of the FFT is characterized by the second-order moments, therefore the cross-spectral matrices contain the complete information of all the statistical properties of the original signals in the frequency domain. All higher-order moments of the FFT are derived from such second-order moments. Furthermore, a cross-frequency analysis is unnecessary since the FFT coefficients are independent between frequencies. Nevertheless, for certain analysis of signals in the time domain, especially those that are focused on non-linear dynamical properties of the temporal evolution or focused on non-stationary signal analysis, higher-order statistical moments may provide useful information. In that sense, not having available the time signals in our dataset may be accounted for as a limitation ruling out the possibility of such interesting dynamical problems requiring analysis in the time domain.

Detailed descriptions of the first wave Cuban EEG normative project can be found in Valdes-Sosa et al. (1989), Valdés et al. (1992), Szava et al. (1994), and Hernandez-Gonzalez et al. (2011).

## Methods

### Sample

The study was performed in Havana, Cuba from 1988 to 1990. The normative sample was obtained by an exhaustive screening of a random sample from a universe of 116,000 inhabitants of Havana, with stringent criteria for inclusion which rejected 65% of the subjects that were recruited. The age range under study was from 5 to 80 years old, although there are two subjects 85 and 97 years old respectively. The subjects were selected through a quasi-random procedure to guarantee an adequate age representation of the whole age span in the sample. The age range was divided into quasi-logarithmically spaced intervals (yearly from 5 to 15.9; every 2 years from 16 to 19.9; every 5 years from 20 to 80) in order to deal with “growth spurts.” In each age group, at least 8 subjects were included, so that the sample size was roughly proportional to the rate of variation of the descriptive parameters. The final normative sample consisted in 211 subjects (105 males, 106 females) healthy subjects in the mentioned age range (Valdés et al., 1992).

### Exclusion Criteria

The study was performed in collaboration with the Cuban government and the Ministry of Public Health of the Republic of Cuba, with the participation of the Family Doctors system in Havana. A populational sample of more than 600 subjects was selected using the quasi-random procedure explained above. The nurses working with the Family Doctors visited the subjects selected in their locality. They explained the subjects the purpose of the study and informed them about all data acquisition protocols as well as safety measures. Then they asked for the subjects' informed consent. They also explained the subjects that they would not receive any monetary compensation for participation, but they would receive full salary at work for the days they have to participate at the study.

To those subjects who gave their consent, the nurses applied an exhaustive clinical history with the purpose of selecting subjects who were functionally healthy. Subjects that did not satisfied those requirements did not continue in the normative study. Those who were identified to may have a possible medical condition were referred to the appropriate specialist.

The exclusion criteria used for this stage are listed in Table 1.

**Table 1:** Exclusions criteria.

**Criteria**	**Description**
Medical conditions	Malignant systemic disease requiring chemotherapy, diabetes, thyroid dysfunction (hyper or hypothyroidism), rheumatic disease, muscular dystrophies, liver cirrhosis, sickle cell anemia, Wilson's disease, lupus erythematosus (SLE), malnutrition, cardiovascular diseases, arterial hypertension, infectious diseases, AIDS, respiratory diseases, history of reactions to medications with hypersensitivity type I, current pregnancy or lactation, permanent metal appliances, cardiac pacemakers or other type of metal in any part of the body (fixed prosthesis, fragments of bullet, etc.)
Neurological disease	Chronic systemic diseases of the Central Nervous System (CNS), seizures or other attacks, malignant expansive processes of the SNC-radiotherapy, more than one loss of consciousness, cerebrovascular accidents, transient cerebral ischemia, migraine or frequent headaches, severe neuropathies, history of cranial trauma with or without loss of consciousness
Psychiatric disorder	History of previous psychiatric treatments, tics, stuttering, mental retardation, suicide attempt, depression, sociopathic behavior, anxiety disorders, panic attacks, schizophrenia, obsessive-compulsive disorder, drug use and abuse, alcoholism, hyperactivity and attention deficit, dementia.
Pre-natal and perinatal antecedents	Difficult pregnancy as indicated by the obstetrician, preterm delivery, gestational hypertension, diabetes, obesity, early membrane rupture in pregnancy, placenta previa, retroplacental hematoma, other infections.
Sleep disorders	Nocturnal terrors, somnambulism and others
Familiar pathological background	Epilepsy, neurodegenerative diseases, multiple sclerosis, Wilson's disease, schizophrenia, manic depressive disorder.
Drug addiction.	Alcohol abuse, non-legal drugs, smoker of more than 20 cigarettes per day, drugs affecting the CNS, direct and maintained exposure to toxic substances such as pesticides, heavy metals, phosphorous organs and organic solvents.
Neurological physical examination	Any abnormality in the neurological physical examination (hypertonia, hypotonia, asymmetry of reflexes, decrease in visual acuity, nystagmus, etc.)

The subjects that were classified as functionally healthy continue to the EEG recording. Expert neurophysiologists visually evaluated the recordings and rejected those ones with abnormalities in the background EEG activity characterized by any of the following: (a) out of range average amplitude, dominant frequency, (b) absence of normal modulation patterns, (c) inadequate anterior/posterior organization according to age, (d) paroxysms, (e) lack of reactivity to eye-opening, hyperventilation or photo stimulation maneuvers.

As a result, 211 healthy subjects (105 males, 106 females) were selected to create the first Cuban Normative database.

### Subjects' Preparation

The subjects were recorded during the morning to guarantee the state of wakefulness. The following instructions were given prior to the EEG recording and checked for just before the session: (a) to go to bed before 11 pm the night before and sleep for at least 8 h; (b) to abstain from alcohol, coffee, black tea, chocolate or soda the day before; (c) to have a normal breakfast in the morning. Additionally, before starting the recording at the clinic they were offered a snack to avoid prolongated fasting period.

### Data Recording and Edition

A digital electroencephalograph (MEDICID-3M) was used with gain of 10 000, filters from 0.3 to 30 Hz and 60 Hz notch, noise 2 mV RMS, sampling period 10 ms. Nineteen silver disc scalp-electrodes were placed according to the International 10/20 System (impedance below 5 KΩ). Recordings were performed with monopolar linked ear reference. Bipolar, average reference and Laplacian montages can be calculated off-line.

Ten to 12 min of EEG were recorded in a dimly lit room, with the subject in resting state, under constant surveillance to prevent drowsiness and in order to control hyperventilation. Samples of EEG were recorded for each subject, in the following states: eyes closed (EC, 5 min) and eyes open (EO, 3 min).

Eye movements and blinking were monitored by electro-oculogram (EOG). Expert electroencephalographers carefully edited the EEG signals and visually selected 24 artifact free segments of 2.56 s duration; 24 segments for EC, EO and HV.

### Transformation to the Frequency Domain

Data analysis sample spectra were calculated by the Fast Fourier Transform (FFT) and cross-segment averaging (Brillinger, 1974). Cross-spectral matrices were calculated for every 0.39 Hz, from 0.39 to 19.11 Hz. As pointed out in Pascual-Marqui et al. (1988) the sample spectra show a considerable degree of smoothness which make them amenable to parametric descriptions.

By means of appropriate transformation matrices (Katznelson, 1981) cross spectral matrices for the linked earlobe reference can be transformed to average reference montage or the Laplacian (Pascual-Marqui et al., 1988).

### Data sharing: Format and Accessibility

The dataset shared in this contribution is publicly available at

Github: https://github.com/oldgandalf/FirstWaveCubanHumanNormativeEEGProject and Zenodo: https://zenodo.org/record/4244765, with doi: 10.5281/zenodo.4244765.

The data contains 198 for Eyes Close and 211 for Eyes Open cross-spectral matrices obtained from the normative sample. The files are compressed as ZIP files, one for Eyes Close (EyesClose.zip) and the second one for Eyes Open (EyesOpen.zip). Inside the ZIP files, the subjects' data are saved in MAT MATLAB format. The name of each file starts with an “A” for Eyes Close and with “B” for Eyes Open, followed by the subject's code and ending with “_cross.mat.”

Inside each MAT file, there are three variables: the subject's age, the sex, the cross spectral matrices for the whole frequency range (MCross), and a vector containing the specific frequency bins in which the cross-spectral matrices were calculated.

MCross is a 3D matrix of 19 × 19 × 49, where 19 is the number of electrodes and 49 is the number of frequencies, equally spanned from 0.39 to 19.11 each 0.39 Hz (“frange” variable). The elements of MCross are real values in the diagonal, for the values of the power spectrum foe each electrode and frequency. Outside the diagonal, the MCross values are complex number containing the cross-spectral values for each pair of electrodes and frequency.

This dataset is accompanied by an XLS file which contains the list of all subjects, including Code, Age, Sex and whether the Eyes Close and Eyes Open data are available.

## Relevant Information

### Normative Data Base Validity

Can a normative database collected in one country be useful to assess EEG deviation of normality of persons from different countries? The transcultural validity of EEG normative data has been stablished by John et al. (1977), Alvarez et al. (1987), Harmony et al. (1990), Chabot et al. (2001), and Prichep (2005). Data from several countries were found to follow the same statistical regularities. No sex or cultural differences were found, only a strong dependence of the EEG spectra with age.

### Usefulness of Normative Data

The normative databases are useful tools for assessing deviations from the expected normal parameters in a population. In the case EEG, it is well-known its dependence with the age. The same parameters which are consider normal for a person of a specific age, may not be normal for a person of different age. To this regard, the normative databases allow calculating regression equations with the age as independent factor, to standardized measurements of “normality deviation,” age dependent in a said population. An example of this of measurement is the *Z* probabilistic of deviation of normality, which is a normalized type of measurement which makes comparable individual differences against normal parameters of subjects with different ages.

The normative database that we are publicly sharing through this work has been successfully used for 30 years to characterize normal and pathological resting sate brain functioning of Cubans and other countries populations (Fernández-Bouzas et al., 1995; Machado et al., 2004; González-Hernández et al., 2005; Taboada-Crispi et al., 2018; Calzada-Reyes et al., 2019). It has a high potential for EEG studies of different nature and may be very useful to characterize brain differences from normality and make the results of different populations comparable.

It may be used to obtain regression equations of both the EEG spectra as well as the cross-covariances between the pairs of electrodes and to study the normal patterns of coherences at the scalp. Of course, estimation of the cross-spectra at the sources can be obtained by means of different inverse methods.

### Suggested EEG Spectra Baseline Standardization

One of the common problems when using EEG data coming from different sources is how to make them comparable. EEGs coming from different devices may be different due to external factors not related to neurophysiology but to hardware differences, amplifiers, noise, and other factors. Additionally, factors like the skull thickness, the hair, skin conductance, etc. may be the source of other differences in the EEG spectra. A random general scale factor (GSF) affecting EEG records due to non-neurophysiological differences has been described in Hernández et al. (1994). It was shown that the maximum likelihood estimate of GSF for an individual is the average of all log power values across all derivations. Correcting the EEG recordings by this factor may help to eliminate those differences among individuals making them more comparable for statistical purposes. In the shared code in GitHub we have included the MATLAB code for calculating the GSF from the cross-spectral matrices like the ones we are sharing. Before calculating the GSF, the procedure involves first re-referencing the data to the Average Reference (Mardia et al., 1997).

### Projected Uses of This Normative Dataset

Besides the previously mentioned advantages for the EEG community of making this dataset publicly available, here we mention some concrete examples of possible uses based on the recent EEG literature. This data set may be used to study the normal development of second order frequency domain properties of the EEG with new methods like:

- Evaluation of preprocessing methods such as improved EEG references as in Hu et al. (2018).
- The creation of age-dependent normative equations, not of isolated element of the Cross-spectral matrices, but rather considering them in their totality as elements of the Riemannian manifold of Hermitian positive definite matrices (Sabbagh et al., 2020).
- To calibrate and test new methods for source localization of activation and connectivity as in He et al. (2019).
- As the basis for the construction of classification models to predict abnormal EEG activity as in Bringas Vega et al. (2019).

### Data Characterization

For illustrative purpose, four age groups were created, which consisted in taking the average of all subjects in different age intervals, as follows:

**Table:** 

**Age group**	***N***
5–7 years-old	15
9–11 years-old	15
20–25 years-old	9
60–80 years-old	35

The individual cross spectra were re-referenced to the average reference and corrected by the Global Scale Factor. Then they were transformed to the logarithm scale to achieve Gaussian distribution. The mean spectra for each group and electrode were calculated across the whole frequency range, both for Eyes Close and Eyes Open.

**Figure 1:**
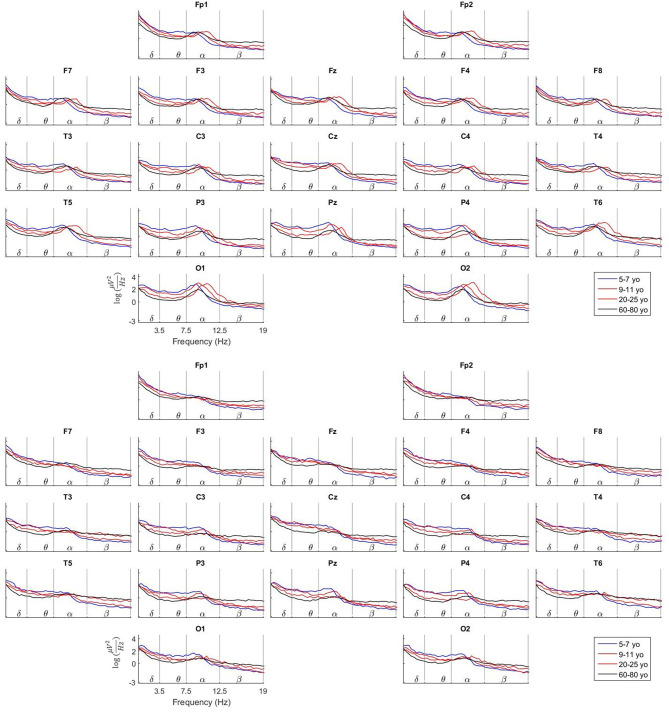
Comparison of the Log average spectra between Eyes Close and Eyes Open for four age groups. These plots illustrate the maturation of the EEG spectra at the scalp with age. Apparent changes can be observed in the behavior of the Alpha peak related to age, head positioning and neurophysiological stage.

Plots of the mean value of the EEG spectra for all electrodes for the four age groups are very informative to assess the validity of our data. The plots are produced both for the Eyes Close and Eyes Open conditions. See Figure 1. In the upper part of the figure, the spectra for the Eyes Close condition is shown, and the spectra for Eyes Open is shown in the lower part.

To highlight just few of the features that can be observed, note that in the Eyes Close condition the Alpha peak is present specially in the occipital and parieto-temporal electrodes while its amplitude is much decreased toward the frontal electrodes. At the same time, the Alpha peak is much flattened, especially in the occipital electrodes in the Eyes Open condition. This is due to the well-known fact of the Alpha activity suppression when the person opens the eyes.

A close look to the occipital electrodes in Eyes Close evidence that the Alpha peak appears at a slower frequency (around 9 Hz) in the group of 5–7 yo. This Alpha peak becomes faster with age to get a maximum frequency of around 10.5 Hz in the group of 20–25 yo. In the group of 60–80 yo the Alpha peak becomes again slower, like the 5–7 yo group. Additionally, note that the amplitude (absolute power) of the EEG spectra in the 60–80 yo group is much smaller than in the younger groups. Many aspects of the EEG maturation can be inferred from the study of the information provided by our normative data.

## Data Availability Statement

The datasets presented in this study can be found in online repositories. The names of the repository/repositories and accession number(s) can be found in the article/Supplementary Material.

## Ethics Statement

The studies involving human participants were reviewed and approved by Committee of Ethics of the National Center for Scientific Research. Written informed consent to participate in this study was provided by the participants' legal guardian/next of kin.

## Author Contributions

JB-B participated in data collection and curation, algorithms programming, and data processing. LG participated in data curation and statistical analysis. EA participated in algorithms programming and data analysis. TV did visual inspection of the data, artifact rejection, and data epoching. PV-S was the director and organizer of the project and participated in data analysis, processing, algorithms programming. All authors contributed to the article and approved the submitted version.

## Conflict of Interest

The authors declare that the research was conducted in the absence of any commercial or financial relationships that could be construed as a potential conflict of interest. The Reviewer VP declared a past co-authorship with one of the Authors PV-S to the handling editor.
